# Fatal airway obstruction following arterial trauma during internal jugular venous cannulation

**DOI:** 10.4103/0972-5229.76085

**Published:** 2010

**Authors:** Aparna Williams, Ashu S. Mathai, Gaurav Bhatia, John Abraham

**Affiliations:** **From:** Department of Anaesthesiology and Critical Care, Christian Medical College, Ludhiana, India

**Keywords:** Airway obstruction, arterial trauma, internal jugular venous cannulation

## Abstract

Central venous cannulations are commonly performed in the intensive care unit. However, these may be associated with severe mechanical or bleeding complications. Here, we describe a patient who died following severe and rapid airway obstruction secondary to an arterial trauma during internal jugular vein cannulation. This case report highlights the importance of prompt recognition of arterial trauma so that it can be repaired surgically instead of sheath removal. The prompt diagnosis of an impending airway obstruction and obtaining early airway access cannot be overemphasized. Finally, we discuss the risk factors associated with this complication and what we could have possibly done to prevent this outcome.

## Introduction

Arterial punctures can occur in 6–25% of internal jugular venous cannulations.[1] However, this complication leading on to fatal airway obstruction is extremely rare and only one case of the same has been reported in literature.[2]

## Case Report

A 65-year-old obese lady [body mass index (BMI) 35 kg/m^2^], with a diagnosis of pyelonephritis, requiring intravenous antibiotics, was shifted to the intensive care unit for central venous cannula (CVC) insertion as her peripheral venous access was poor. Earlier investigations had revealed mildly deranged bleeding parameters as follows: prothrombin time of 13 with a control of 12, activated partial thromboplastin time of 41.4 with a control of 25, platelet count of 200,000/mm^3^ and a hematocrit of 27/mm^3^.

After transfusion of four units of fresh frozen plasma, a landmark-based right internal jugular venous (IJV) cannulation was attempted. Using the high central approach, the IJV was punctured and free-flowing dark “venous” blood was aspirated. After guidewire insertion, the vein was dilated. However, the 7-Fr triple lumen catheter could not be negotiated, and hence, the cannula over guidewire was removed. External pressure was applied over the puncture site for 5 minutes and, before the next attempt could be made, the patient was noticed to be agitated. The drape covering her head was removed and the patient was found to be diaphoretic, pale and tachypneic. Immediately, all the drapes were removed and a massive anterior neck swelling was evident, associated with breathing difficulty. Her pulses were feeble and heart rate was 145/minute and oxygen saturation by pulse oximetry was 80–85%. She was initiated on oxygen at fiO_2_ 60%. In view of an impending airway obstruction, an immediate attempt was made to intubate the trachea, which was unsuccessful due to the severely distorted internal anatomy and secretions in the airway. The ENT surgeon was notified for an urgent tracheostomy. Intravenous crystalloids and colloids were rapidly transfused. Meanwhile, the anterior neck swelling was increasing rapidly in size and the patient’s heart rate was 160/minute. The ENT surgeons present began doing an emergency tracheostomy while bag and mask ventilation was carried out. A second attempt at laryngoscopy using the BURP (backwards, upwards and right posterior) maneuver barely revealed the posterior part of the glottic opening, and a size-6.5 endotracheal tube was successfully slipped in, just as the skin and subcutaneous tissues were being opened. A large hematoma was found extending over the entire anterior surface of the neck. The patient developed bradycardia with a heart rate of 30/minute which quickly deteriorated to asystole. Cardiopulmonary resuscitation was immediately instituted but despite 40 minutes of advanced cardiac life support therapy, the patient could not be resuscitated. The time from initial vascular puncture to cardiac arrest was less than 20 minutes.

## Discussion

A review of literature suggests that although arterial trauma is not uncommon during central venous cannulations (CVCs),[1] a fatal outcome secondary to severe and rapid airway obstruction such as this is very rare. Our patient had severe airway obstruction associated with hemodynamic instability, and the presence of a large hematoma over the anterior surface of the neck. Hence, the most likely cause of this complication would have been trauma following dilator injury to the carotid artery, resulting in rapid blood loss and formation of hematoma which caused fatal airway obstruction.

Carotid artery trauma has been reported in 6–25% of patients, following landmark-based internal jugular venous cannulations.[1] The other, rarer arterial punctures include those involving the subclavian artery (0.1–1% of IJV cannulations)[1] and the aorta, which is associated with a 90% death rate.[3] Despite the advent of ultrasound guided vascular cannulation, which has reduced the incidence of complications drastically,[4] many centers in our country still rely on the landmark-based technique for cannulation, which has a success rate of 75–99%.[1] The factors associated with bleeding and mechanical complications during CVC insertions include unsafe manipulation, kinking of the guide wire, operator inexperience, increased needle passes, severe dehydration, morbid obesity, short neck, emergency procedures and coagulopathy.[5] Our patient had some of these risk factors, viz., obesity, short neck and coagulopathy.

The key factor that determines survival is prompt recognition of the arterial trauma. We failed to recognize the arterial trauma and pulled out the dilator directly, a practice that needs to change and henceforth be strongly discouraged, especially if the artery has been dilated or the cannula inserted. The removal of the CVC and application of pressure over the arterial puncture site is reportedly associated with a significant risk of hematoma, airway obstruction, stroke, and even death.[6] Hence, if an arterial trauma is recognized during CVC insertion, the recommended approach is to leave the sheath in place and refer the patient for early and safe surgical arterial repair.[7]

In previous case reports of severe airway obstruction following inadvertent arterial trauma during IJV cannulation, obtaining timely airway access was the most important factor which determined survival.[2,8,9] In our patient, there was a delay in recognizing the early signs of airway compromise due to the drapes covering the patient. Also, the cessation of external bleeding when pressure was applied led us to falsely believe that the bleeding was controlled. By the time the airway obstruction was recognized, the hematoma had increased significantly and visualization of the larynx was impossible. Thus, there was a delay by nearly 10–12 minutes before the airway was finally secured.

In conclusion, arterial trauma during IJV cannulation can lead to catastrophic consequences if not rapidly detected. The factors which might have helped us avoid this unfortunate outcome are the use of transparent drapes through which we could have noted the developing hematoma earlier, better airway preparedness, and possibly, the use of ultrasound guidance for cannulation, more so with the risk factors involved in our patient. Also, if the arterial trauma had been recognized, especially after dilatation, the correct approach would have been to leave the dilator in place while referring the patient for emergent and safe surgical repair.
